# Mental Health Status of Healthcare Professionals and Students of Health Sciences Faculties in Kuwait during the COVID-19 Pandemic

**DOI:** 10.3390/ijerph18042203

**Published:** 2021-02-23

**Authors:** Zahra Alsairafi, Abdallah Y. Naser, Fatemah M. Alsaleh, Abdelmoneim Awad, Zahraa Jalal

**Affiliations:** 1 Department of Pharmacy Practice, Faculty of Pharmacy, Kuwait University, Kuwait City 12037, Kuwait; fatemah.alsaleh@ku.edu.kw (F.M.A.); abdelmouneim.awadhussein@ku.edu.kw (A.A.); 2 Department of Pharmaceutical Sciences and Clinical Pharmacy, Isra University, Amman 33, Jordan; abdallah.naser@iu.edu.jo; 3 School of Pharmacy, Institute of Clinical Sciences, University of Birmingham, Edgbaston B15 2TT, UK; z.jalal@bham.ac.uk

**Keywords:** coronavirus disease, COVID-19, mental health, healthcare professionals, students, depression, anxiety, survey, Kuwait

## Abstract

Objectives: This study aimed to assess the impact of the COVID-19 pandemic on the mental health status of healthcare professionals (HCPs) and undergraduate students in the health sciences center (HSCUs). In addition, it explored the factors associated with the increased levels of mental health burden among the study population. Methods: A cross-sectional study was performed using two online-administered questionnaires: the Patient Health Questionnaire (PHQ-9) and the Generalized Anxiety Disorder 7-item (GAD-7), which were distributed in parallel to HCPs and HSCUs in Kuwait. These instruments are validated assessment scales to assess mental health status: depression (PHQ-9) and anxiety (GAD-7). Statistical analyses were carried out using SPSS- version 25. Results: A total of 857 individuals (559 HCPs and 298 HSCUs) participated in this study. The prevalence of moderately severe depression or severe depression (PHQ-9 total score of ≥15) among respondents was 66.6%. The median (interquartile range, IQR) PHQ-9 score was significantly higher among HSCUs (20 {11.5}) compared to HCPs (17 {8}). The prevalence of severe anxiety (GAD-7 total score of ≥15) among respondents was 36.7%. There were no significant differences between the median (IQR) GAD-7 scores among the HCPs (14 {7}) and HSCUs (13 {8}). Binary logistic regression analysis revealed that three variables were significantly and independently associated with severe depression among HCPs. The prevalence of severe depression was found to be greater among females compared to males. In addition, it was significantly lower among those who were aged ≥50 years, and those who reported that they were not in direct contact with COVID-19 patients. Among HSCUs, females showed greater depression than males. In contrast, those aged >29 years and who had no history of chronic disease showed lower depression compared to their counterparts in the 18–29 years age group and who had a chronic disease history. Conclusions: The COVID-19 pandemic had a significant negative impact on the mental health of HCPs and HSCUs in Kuwait. This highlights the need for proactive efforts to support their mental health and well-being through educational campaigns and psychological support programs.

## 1. Introduction

The coronavirus disease 2019 (COVID-19) is an illness caused by a novel beta-coronavirus, named the severe acute respiratory syndrome coronavirus 2 (SARS-CoV-2). It appeared firstly in Wuhan, China in December 2019, and then spread rapidly worldwide, leading to a fatal pandemic. SARS-CoV-2 infection appears to cause a wide range of symptoms, including asymptomatic infection, mild infections of the upper respiratory tract, severe viral pneumonia, respiratory failure, multiple organ failure, and death. Mortality rates are higher among older adults, particularly those with chronic diseases [1]. Due to its highly contagious nature, it is transmitted through respiratory droplets and close contact with infected people [2]. Consequently, governments have taken strict measures such as isolating infected humans and their family members, cancelling public transportation, implementing travel restrictions, curfews and social distancing, and obligating people to wear face masks [3]. In Kuwait, as of 1 February 2021, a total of 165,257 diagnosed cases of COVID-19 had been reported, with 959 deaths, 157,931 recovered cases, and 6367 cases undergoing treatment [4]. Since the identification of the first few cases of COVID-19 in Kuwait in February 2020, the government applied precautionary measures similar to those seen globally to prevent the spread of the disease. In addition to the country lockdown, the Kuwaiti government applied strict discipline to people violating the imposed measures, such as 3 months jail and fines of 30,000 Kuwaiti dinars (USD 98,610).

The public healthcare system in Kuwait is divided into primary, secondary, and tertiary care. Primary care is delivered through general and specialized polyclinics distributed across five healthcare regions. Secondary care is provided through six general hospitals, and tertiary care is delivered through fifteen specialized centers [5]. During the COVID-19 outbreak, some of the healthcare settings operated regularly, while others provided units for the management of patients with COVID-19. The Ministry of Health (MOH) also imposed that all healthcare professionals (HCPs) are obliged to be on duty and prohibited any leave during this ongoing pandemic, in order to control the situation and prevent the collapse of the healthcare system. The health sciences center of Kuwait University includes five faculties: medicine, dentistry, pharmacy, allied health sciences, and public health. During the COVID-19 outbreak, education at Kuwait University was suspended (from February until July 2020) and no alternate measures were implemented to resume education during this period. It has been proposed that such restrictive measures would have resulted in short- and long-term adverse consequences, such as stress-related psychiatric symptoms like anxiety and depressive mood among the affected people [6].

The sudden surge of increased work at the treatment front-line, the threat of being infected and transmitting the virus to family members, and the consequent death or illness of a relative or friend due to the pandemic could have had a psychological impact on people working in the health sector, especially the HCPs who have direct or indirect contact with COVID-19 patients [7,8,9]. In addition, this pandemic could have had a negative impact on university students who lived with a high degree of pressure and fears about the future due to the suspension of education [10]. In this context, recent studies have shown that the global growth in the number of cases and deaths, the collapse of the healthcare system in many countries, and the subsequent lack of effective medical treatment are factors prompting public fear, anxiety, and/or depression, which is usually neglected during pandemic management [11,12,13]. Thus, studying the psychological wellbeing of populations during the COVID-19 pandemic is paramount. This is particularly the case in Kuwait, where around one-quarter and one-third of the general public showed anxiety and depressive symptoms during this pandemic, respectively [14], and the majority of students (90.9%) at Kuwait University reported moderate to high life stress [15]. Therefore, this study aimed to assess the impact of the COVID-19 pandemic on the mental health status of people who are at high risk of being affected. For example, it is vital to evaluate the mental health burden on HCPs who experienced increased working hours, feelings of incapability, and self-isolation from families. Unlike the general public, people working in the health sector perceive a risk of acquiring the virus at work due to suspected cases and, hence, are more likely to experience depression and anxiety [14]. As future health professionals, this study also included undergraduate students in the health sciences center (HSCUs). In addition, this study explored the factors associated with the increased levels of mental health burden among the study population, in order to recommend targeted interventions and support.

## 2. Materials and Methods

### 2.1. Study Area and Design

Kuwait is a Middle Eastern country with an area of 17,820 km^2^ and a population of 4,301,539 people (2020 estimate) [16]. A quantitative, prospective, and cross-sectional study was performed to assess the mental health status of HCPs and HSCUs during the COVID-19 outbreak. It was conducted between May and July 2020. Ethical approval for this study was obtained from the Human Ethical Committee, Health Sciences Centre, Kuwait University (VDR/EC/3655).

### 2.2. Study Population

The sample size was estimated based on the World Health Organization (WHO) recommendations for the minimal sample size needed for a prevalence study [17]. Using a confidence interval of 95%, a standard deviation of 0.5, and a margin of error of 5%, the required sample size was 385 participants. A convenience sample of eligible participants (HCPs and HSCUs) was invited to participate in the study. Data were collected using two online-administered questionnaires sent through various social media platforms (e.g., Twitter and WhatsApp). Each study population was invited using a specific survey-link (e.g., one for the HCPs and another for HSCUs). All HCPs (*n* = 9863) who work at the six generalized hospitals, which provided units for COVID-19 patients during the pandemic, were invited [18]. The hospitals were approached by the researcher. The head of each department (e.g., medical/pharmacy/nursing) in these hospitals were visited to help in obtaining WhatsApp addresses of HCPs and distributing the survey-link to them. For HSCUs, the researcher used the official Twitter address of the HSC of Kuwait University to invite them to participate in the study. In addition, one staff member at each faculty invited students in his/her contact list through WhatsApp messages. The inclusion criteria were an age of ≥18 years, living in Kuwait during the pandemic period, and working in governmental hospitals that were operated for the management of COVID-19 patients for HCPs, and studying at HSC of Kuwait University for HSCUs. The study objectives, maintenance of confidentiality, and anonymity were clearly explained at the beginning of the survey. A reminder message was sent to all eligible participants before the end of the data collection period (on 1st of July). All participants voluntarily participated in the study and gave written consent.

### 2.3. Study Questionnaires

Previously validated assessment scales, the Patient Health Questionnaire (PHQ-9) and the Generalized Anxiety Disorder 7-item (GAD-7) were used to assess depression and anxiety among the study’s participants. The study instruments have been used among different populations to assess their mental health status [19,20,21,22]. The PHQ-9 and GAD-7 instruments asked the participants about the degree of applicability of each item (question), using a 4-point Likert scale. Participants’ responses ranged from 0 to 3, where 0 means “Not at all” and 3 means “Nearly every day”. The PHQ-9 is a self-report questionnaire that is commonly used as a screening tool for depression [23]. It is a 9-question instrument to assess the presence and severity of depression [24,25]. Items are scored from 0 to 3 generating a total score ranging from 0 to 27. A total score of 0–4 indicates minimal depression, 5–9 mild depression, 10–14 moderate depression, 15–19 moderately severe depression, and 20–27 severe depression [26]. The GAD-7 instrument includes 7 items, and was initially developed to screen for GAD, and also proved to have good sensitivity and specificity as a screening tool for anxiety, panic, and social disorders [27,28]. Items are scored from 0 to 3 generating a total score ranging from 0 to 21. A total score of 0–4 indicates minimal anxiety, 5–9 mild anxiety, 10–14 moderate anxiety, and 15–21 severe anxiety [29].

The prevalence rates of depression and anxiety were determined using a cut-off point as recommended by the authors of the PHQ-9 and GAD-7 scales. In this study, depression was defined as a total score of 15 or more, which indicates moderately severe or severe depression. Anxiety was defined as a total score of 15 or more, which indicates severe anxiety. The rates were estimated by dividing the number of participants who exceeded the borderline score (15) by the total number of participants in the same population. In addition, demographic information was collected from the participants, such as age, gender, and marital status. All participants were asked whether they were worried about being infected with COVID-19 or transmitting it to family members (yes/no question). Moreover, HCPs were asked about whether they were in direct contact and/or were providing direct or indirect medical care for patients with COVID-19 during this pandemic. Students were asked about their years of study and history of chronic diseases. The questionnaires were pre-tested for content, design, readability, and comprehension on two HCPs and three HSCUs, and minor modifications were included.

### 2.4. Data Analysis

Descriptive statistics were used to describe participants’ demographics. Continuous data were reported as median (interquartile range, IQR). Categorical data were reported as percentages (95% confidence interval, CI). Comparative analysis was performed on the depression and anxiety scores across different demographic and other characteristics of respondents using Mann-Whitney and Kruskal–Wallis tests because the data were not normally distributed based on the Shapiro–Wilk and Kolmogorov–Smirnov tests for normality. Logistic regression was used to estimate odds ratios (ORs) with 95% CIs. Logistic regression models were carried out using anxiety or depression scores above the cut-off points for severe cases. A two-sided *p* < 0.05 was considered statistically significant. The statistical analyses were carried out using the Statistical Package for Social Sciences (IBM Corp, Armonk, NY, SPSS Statistics for Windows, version 25).

## 3. Results

### 3.1. Participants Characteristics

A total of 857 responders (HCPs = 559 and HSCUs = 298), 18 years and above, who fully completed the questionnaire were included in the statistical analysis. Table 1 shows the characteristics of the study’s participants. The majority (*n* = 633, 73.9%) were females, married (*n* = 425, 49.6%), and with regards to the HCPs, most had a high income (*n* = 267, 47.8%). Physicians contributed to the largest proportion of the participating HCPs (*n* = 213, 38.1%) followed by pharmacists (*n* = 189, 33.8%), allied health professionals (*n* = 127, 22.7%), and nurses (*n* = 30, 5.4%). Most of the HCPs (*n* = 478, 85.5%; 95% CI: 82.3–88.3) reported that they were in direct contact with COVID-19 patients during the pandemic period. In relation to HSCUs, around 33.6% (*n* = 100) were in their early years, 48% (*n* = 143) in the middle years, and 18.4% (*n* = 55) in their final years. Only 15.4% (*n* = 46; 95% CI: 11.6–20.2) of HSCUs reported a history of chronic disease. The majority of the participants (*n* = 549, 64.1%; 95% CI: 60.7–67.3) reported that they were concerned about contracting COVID-19 or transmitting it to their family members.

### 3.2. Prevalence of Depression and Anxiety

Table 2 shows the prevalence of depression and anxiety among the respondents stratified by their severity. Two-thirds (*n* = 571; 66.6%; 95% CI: 62.9–69.3) were found to have PHQ-9 scores ≥15, which indicates moderately severe depression or severe depression. The prevalence of mild, moderate, moderately severe, and severe depression among respondents were 3.2%, 28.2%, 35.1%, and 31.5%, respectively. Over one-third (*n* = 314; 36.7%; 95% CI: 79.4–84.6) had GAD-7 scores ≥15, which indicates severe anxiety. The proportions of mild, moderate, and severe anxiety among respondents were 17.8%, 45.5%, and 36.7% respectively.

### 3.3. Comparison of Depression and Anxiety Scores across Different Characteristics of Respondents

Table 3 presents the median (IQR) scores of depression and anxiety stratified by the participants’ characteristics. The median (IQR) PHQ-9 score for depression was significantly higher among HSCUs (20 {11.5}) compared to HCPs (17 {8}); *p* = 0.006 and females (18 {9}) compared to males (15 {8}); *p* < 0.001. In HCPs, the median (IQR) PHQ-9 score for depression was significantly higher among those aged 18–29 years (19 {10}) and 30–49 years (17 {8.25}) compared to those aged ≥50 years (15 {8.20}), *p* < 0.05. In HSCUs, the median (IQR) PHQ-9 score for depression was significantly higher among those aged 18–29 years (20 {11.75}) and students with a history of chronic disease (23.5 {8}) compared to those aged >29 years (18 {11.70}), *p* = 0.001, and those with no chronic disease history (18 {10}), *p* = 0.018, respectively. The median (IQR) GAD-7 scores for anxiety were significantly higher among females (14 {7}) compared to males (12 {8}), *p* < 0.001, and HSCUs with a history of chronic disease (16.5 {2.75}) compared to those with no history of chronic disease (12 {7}), *p* = 0.017. Interestingly, both populations in the age range of 18–29 years had comparable median depression and anxiety scores. The two populations had the same median anxiety score (13), whereas HSCUs showed higher median depression scores (20 {11.75}) compared to HCPs (19 {10}), *p* < 0.05.

In addition, those who worried about being infected with COVID-19 or transmitting it to their family members had higher median depression and anxiety scores. Pharmacists and HCPs with high-income levels showed lower median depression scores. Students in their final years had the least median depression and anxiety scores compared to their colleagues in other year levels. However, there were no significant associations between all those variables.

### 3.4. Factors Independently Associated with the Increased Levels of Mental Health Burden

Using logistic regression, among HCPs, the following group were identified to be at higher risk of depression: females, younger than 50 years, who reported that they were in direct contact and provided medical care to COVID-19 patients during the pandemic period (Table 4). Among HSCUs, females who are >29 years were at higher risk of depression, and those who reported a chronic disease history were at higher risk of depression and anxiety (Table 5).

## 4. Discussion

The pivotal role of HCPs during pandemics as front-liners is fundamental, and makes them more vulnerable to depression, anxiety, and stress due to overwhelmed healthcare systems and fear of being infected or transmitting the virus to their families [8,30,31,32,33,34,35,36,37,38]. Psychological impairment would significantly affect the attention, cognitive functioning, and clinical decision-making of HCPs [39]. Previous studies have also suggested that public health emergencies had a major psychological impact on university students expressed as anxiety, fear, and worry [10,40,41]. The main aim of the current study was to assess the impact of the COVID-19 pandemic on the mental health status of HCPs and HSCUs in Kuwait. In addition, this study explored the factors associated with the increased levels of mental health burden among the study population. It was found that the mean depression scores were significantly higher among HSCUs compared to those of HCPs. However, there were no significant differences in the median anxiety scores between the two populations. The high rates of depression and anxiety reported among this population are alarming, and appropriately targeted interventions are needed.

### 4.1. Mental Health Status of HCPs

In Japan and Singapore, during the Severe Acute Respiratory Syndrome (SARS) outbreak, fear and anxiety were prevalent among more than half of HCPs [42,43]. In Greece, nearly half of the HCPs experienced moderately high levels of worry during the H1N1 influenza pandemic [44]. In the case of COVID-19, due to its unclear dynamics, rapidity of spread, high morbidity and mortality rates, and the lack of definitive therapy and preventive vaccines, feelings of fear and stress might be exacerbated, contributing to the pressure and anxiety to HCPs [2,6,45]. In China, half of HCPs were identified to suffer from at least mild depression, 45% with moderate or severe anxiety, and one-third with insomnia during the COVID-19 outbreak [46]. HCPs in Oman had high anxiety rates, where 65% suffered from mild, moderate, or severe anxiety during this recent pandemic [47]. In India, HCPs also showed depression and anxiety symptoms (47% and 50%, respectively) during the outbreak of COVID-19 [48]. Consistent with the previous studies, this study found that more than half of HCPs experienced depression and 37.4% experienced anxiety. However, this was not the case in Saudi Arabia, where only 15% of HCPs reported fear and anxiety or considered rescheduling their duty to avoid contact with COVID-19 patients [38]. This could be attributed to the fact that in Saudi Arabia HCPs passed through several campaigns as a part of preparedness for the upcoming pandemic, to improve seasonal influenza uptake during their previous Middle East Respiratory Syndrome (MERS-COV) outbreak. In Jordan, HCPs reported lower depression and anxiety rates compared to HCPs in Kuwait [49]. This could be related to the fact that in Jordan, HCPs experienced fewer cases of COVID-19 due to the rapid proactive measures taken by the government. In Italy, only 20% of HCPs had depression symptoms and 8% had forms of anxiety during the recent COVID-19 outbreak [50]. Mental health responses to epidemics may differ due to several issues including the availability of clinical evidence, media reports, case and death rates, and the implemented isolation policies [2,34].

### 4.2. Factors Associated with Depression and Anxiety among HCPs

Unsurprisingly, this study showed that depression and anxiety were more prevalent among HCPs who were in direct contact with confirmed/suspected COVID-19 patients. Similarly, studies during the SARS outbreak and COVID-19 reported more psychiatric symptoms among HCPs who were directly exposed to patients [11,47,51,52,53]. Being isolated or quarantined could provoke lethargy among HCPs [6]. Furthermore, Temsah et al. (2020) reported a small percentage of anxiety and anger feelings (7.6% and 16.6% respectively) among HCPs who worked in quarantines. In regard to the specialty of the HCP, it has been proven in previous studies that during pandemics doctors showed a lower level of anxiety compared to other HCPs [8,46,50]. Although the current findings showed slight differences in depression and anxiety rates, doctors reported lower levels of mental distress compared to other HCPs. However, results reported from India were in opposition those of all of the previous studies, and showed no differences between doctors and nurses in their mental distress level during the pandemic [48].

Regarding the age of HCPs, this study reported similar results to those found in Jordan and other countries, where young HCPs were more psychologically affected than others [12,47,48,49,54]. This could be attributed to the fact that young HCPs may have less experience or they may have such fear because they might be parents of children, and young people who need more attention and effort in improving their knowledge about the preventive measures. In comparison, older HCPs may have more experience, better coping skills, and greater knowledge about pandemics and how to deal with such emergencies, even though older people with comorbidities are particularly vulnerable to worse outcomes from COVID-19, which generate extensive worry and anxiety amongst the elderly. The level of education, training, experience, and knowledge about infectious diseases and their management could positively impact the psychological wellbeing of HCPs [55,56]. Although this was not investigated in the current study, belief in the preventive and control measures applied by the MOH could also contribute to the lower rates of depression and anxiety among HCPs [35]. Similar to recent studies, the current study reported that the female gender was a risk factor for psychological distress among HCPs during pandemics [2,46,47,49,50,54,57]. This could be attributed to the increased frequency of hormonal fluctuation in women, which leads to mood changes compared to men [58,59]. However, Chen and colleagues (2020) and Suryavanshi and colleagues (2020) reported no significant differences in depression and anxiety between male and female HCPs.

### 4.3. Factors Contributed to the Mental Health Status of HSCUs

Due to the abrupt suspension of education, with no immediate alternative plans to resume lessons, HSCUs showed high depression and anxiety rates. Students might have major concerns regarding the impact of the current emergent situation on their future education and employment [2,60]. In addition, students’ psychological impairment could be related to the fact that during quarantine there was an absence of interpersonal communication [61,62]. Alternatively, it could be that social media use increased among students due to greater availability of free time during the lockdown, resulting in a negative affect via the updated COVID-19 news and cases. In the context of depression, the current study reported higher rates (72.2%) than Jordan (38.5%) and Iran (27.6%) [41,49]. This could be related to the fact that in Jordan and Iran the universities used remote learning methods instantly, whereas in Kuwait, it took several months to decide on continuing education through distance learning. Uncertainty regarding the future and the disruption of daily routine might contribute to high depression rates [63]. Regarding anxiety, this study also reported higher results (35.2%) than Jordan and China, where university students reported anxiety rates of 21.5% and 24.9%, respectively [10,44]. However, medical students in Iran had comparable results for anxiety rates (38%) to students in Kuwait [41].

In this study, all HSCUs who reported a history of chronic disease showed higher depression and anxiety scores compared to their counterparts who do not have a chronic disease. Unsurprisingly, this result could be due to the awareness of the students about the association between the severity of COVID-19 complications and the presence of chronic diseases [64]. Although the type of chronic disease was not investigated in this study, other studies reported a significant association between history of mental illness and anxiety during COVID-19 outbreak among university students [10,65,66]. Female students showed higher depression and anxiety rates, which is consistent with results reported in Iran [41]. A previous study in Kuwait also reported a significant difference in stress rates among female compared to male students [15]. Such findings may indicate that males and females respond variously to stressors. However, in contrast to results reported from China and other countries, male and female students showed no significant differences in their distress level during the COVID-19 outbreak [10,67].

### 4.4. Implications for Future and Practice

The findings of this study highlighted the impact of the COVID-19 pandemic on the psychological wellbeing of a very important group of the population such as HCPs (who work as front-liners) and HSCUs (who have the ambition to graduate and practice their knowledge in the health sector). These results should raise the awareness of policymakers to propose appropriate interventions that ensure the psychological wellbeing of targeted individuals during the pandemic period. Regular and intensive training for all HCPs on the readiness for pandemics is necessary to improve the experience, skills, and mental wellbeing of HCPs, and to help effectively manage critical situations during the pandemic [64,68]. Although the MOH was highly effective in managing manpower and workforce, and in providing medical supplies and personal protective equipment, during the outbreak, emotional support for HCPs is paramount [44,69]. Psychological support is also crucial for HSCUs, and the university should provide students with psychological assistance, such as a hotline service with psychiatrists to provide them with stress relief activities [11,40,70]. Future qualitative studies are required in this field to help in further exploring and addressing the needs of HCPs and HSCUs during pandemics. In addition, future research could also be conducted to compare depression and anxiety levels among HCPs who work for the management of COVID-19 patients and others who work in other settings that operate regularly during the pandemic.

### 4.5. Strengths and Limitations

To our knowledge, this is the first study in Kuwait that investigated the prevalence of depression and anxiety during the COVID-19 pandemic among important population groups (HCPs and HSCUs). It included HCPs from different specialties such as physicians, nurses, and pharmacists working in the governmental hospitals in Kuwait. In addition, data were collected during the peak of the COVID-19 pandemic, which reflects the collection of the most realistic and valid results. Furthermore, the use of previously validated assessment tools is another strength of the study.

However, there are some limitations. Limited studies have discussed the impact of this novel pandemic on mental health globally and in the Middle East, which limited the comparison of this study’s findings with others. In addition, the use of an online survey for data collection may introduce some bias, where some targeted populations may have been missed, or may lead to a low response rate. However, the reason for the low response rate among HCPs could be time constraints and heavy workloads due to the current situation of the pandemic, and it was unclear why some students did not participate. Because of the cross-sectional design, potential changes in depression and anxiety levels over time could not be measured. In addition, this study assessed the prevalence of acute depression and anxiety during the pandemic period, and whether the participants had chronic depression and anxiety was not investigated.

## 5. Conclusions

The COVID-19 pandemic introduced a critical level of depression and anxiety among the population. However, some groups were intensely affected due to their work, such as HCPs, or due to the suspension of education and the potential disruption to their future, such as university students. Among HCPs, those who were in direct contact with COVID-19 patients and who reported worrying about being infected or transmitting the infection to their families had higher median depression and anxiety scores. In particular, females younger than 50 years were more psychologically affected than others. Among HSCUs, young females with a history of chronic disease were more likely to experience depression and anxiety compared to others. Therefore, to alleviate the psychological impairment of targeted groups of people, hospitals are encouraged to conduct educational campaigns targeting HCPs to improve their knowledge and awareness about COVID-19, and reassure them about the effectiveness of the legitimate prevention and control measures that the MOH applied in providing a safe environment. In addition, HSCUs should be aware and updated about the methods that the university is planning for the continuation of education within the pandemic circumstances. Although on-line courses were targeted at students about remote learning resources, psychological interventions and pastoral support is needed to effectively and appropriately regulate students’ emotions and avoid any losses during this public health emergency.

## Figures and Tables

**Table 1:** Characteristics of the study participants.

Characteristics	Overall (*n* = 857) Frequency (%)	HCPs (*n* = 559) Frequency (%)	HSCUs (*n* = 298) Frequency (%)
Gender			
Males	224 (26.1)	193 (34.5)	31 (10.4)
Females	633 (73.9)	366 (65.5)	267 (89.6)
Age (Years) for HCPs			
18–29	128 (22.9)	128 (22.9)	
30–49	355 (63.5)	355 (63.5)	
≥50	76 (13.6)	76 (13.6)	
Age (Years) for HSCUs			
18–29	289 (97.0)		289 (97.0)
>29	9 (3.0)		9 (3.0)
Marital status			
Single	410 (47.8)	148 (26.5)	262 (87.9)
Married	425 (49.6)	393 (70.3)	32 (10.7)
Divorced/Widowed	22 (2.6)	18 (3.2)	4 (1.4)
Stage of study for HSCUs			
Early years	100 (33.6)		100 (33.6)
Middle years	143 (48.0)		143 (48.0)
Final years	55 (18.4)		55 (18.4)
Monthly income for HCPs			
Low (<1000 KWD)	175 (31.3)	175 (31.3)	
Medium (1000–1500 KWD)	117 (20.9)	117 (20.9)	
High (>1500 KWD)	267 (47.8)	267 (47.8)	
Specialty for HCPs			
Physicians	213 (38.1)	213 (38.1)	
Pharmacists	189 (33.8)	189 (33.8)	
Nurses	30 (5.4)	30 (5.4)	
Allied health professionals	127 (22.7)	127 (22.7)	
Chronic disease history for HSCUs			
Yes	46 (15.4)		46 (15.4)
No	252 (84.6)		252 (84.6)
In direct contact with COVID-19 patients for HCPs			
Yes	478 (85.5)	478 (85.5)	
No	81 (14.5)	81 (14.5)	
Worried about being infected with COVID-19 or transmitting it to family members			
Yes	549 (64.1)	503 (90.0)	46 (15.4)
No	308 (35.9)	56 (10.0)	252 (84.6)

**Table 2:** Prevalence of depression and anxiety among the participants stratified by severity.

Levels of Depression and Anxiety	Overall (*n* = 857) Frequency (%)	HCPs (*n* = 559) Frequency (%)	HSCUs (*n* = 298) Frequency (%)
Depression assessment No. (%)			
Minimal depression	17 (2.0)	0	17 (5.7)
Mild depression	27 (3.2)	23 (4.1)	4 (1.3)
Moderate depression	242 (28.2)	180 (32.2)	62 (20.8)
Moderately severe depression	301 (35.1)	199 (35.6)	102 (34.2)
Severe depression	270 (31.5)	157 (28.1)	113 (38.0)
Anxiety assessment No. (%)			
Minimal anxiety	0	0	0
Mild anxiety	153 (17.8)	109 (19.5)	44 (14.8)
Moderate anxiety	390 (45.5)	241 (43.1)	149 (50.0)
Severe anxiety	314 (36.7)	209 (37.4)	105 (35.2)

**Table 3:** Median (interquartile range, IQR) scores of depression and anxiety stratified by participants’ characteristics.

Variable	Depression Score			Anxiety Score		
	Median	IQR	*p*-Value	Median	IQR	*p*-Value
Population						
HCPs	17.00	8.00	0.006	14.00	7.00	0.143
HSCUs	20.00	11.50		13.00	8.00	
Gender						
Males	15.00	8.00	<0.001	12.00	8.00	<0.001
Females	18.00	9.00		14.00	7.00	
Age (Years) for HCPs						
18–29	19.00	10.00	0.000	13.00	7.75	0.669
30–49	17.00	8.25		14.00	7.00	
≥50	15.00	8.20		12.00	10.00	
Age (Years) for HSCUs						
18–29	20.00	11.75	0.001	13.00	8.00	0.661
>29	18.00	11.70		12.00	8.75	
Marital status						
Single	19.00	10.00	0.085	13.00	8.00	0.716
Married	17.00	9.00		14.00	8.00	
Divorced/Widowed	18.00	13.00		14.00	9.00	
Stage of study for HSCUs						
Early years	19.5	10.50	0.917	14.00	8.00	0.754
Middle years	21.00	12.00		16.00	11.00	
Final years	18.00	7.75		14.00	8.75	
Monthly income for HCPs						
Low (<1000 KWD)	18.00	10.00	0.195	13.50	7.25	0.696
Medium (1000–1500 KWD)	18.00	10.00		14.00	7.00	
High (>1500 KWD)	17.00	9.25		14.00	8.00	
Specialty for HCPs						
Physicians	18.00	10.00	0.326	13.00	7.00	0.631
Pharmacists	17.00	8.00		14.00	8.00	
Nurses	18.00	8.50		13.00	7.75	
Allied health professionals professional	18.00	9.00		14.00	8.00	
Chronic disease history for HSCUs						
Yes	23.50	8.00	0.018	16.50	2.75	0.017
No	18.00	10.00		12.00	7.00	
Worried about being infected with COVID-19 or transmitting it to family members						
Yes	16.00	8.00	0.809	14.00	7.00	0.650
No	15.50	6.75		13.00	9.50	

**Table 4:** Logistic regression analysis for healthcare professionals (HCPs).

Variable	OR (95% CI) for Depression	OR (95% CI) for Anxiety
Gender		
Males (Reference)	1.00	1.00
Females	1.98 (1.34–2.95) (*p* < 0.01)	1.45 (0.97–2.18)
Age (Years)		
<50 (Reference)	1.00	1.00
≥50	0.54 (0.32–0.92) (*p* = 0.001)	0.86 (0.49–1.50)
Marital status		
Single (Reference)	1.00	1.00
Married	0.89 (0.59–1.35)	0.81 (0.54–1.22)
Divorced/Widowed	1.15 (0.39–3.43)	1.71 (0.63–4.64)
Monthly income		
Low (<1000 KWD) (Reference)	1.00	1.00
Medium (1000–1500 KWD)	1.39 (0.86–2.24)	1.30 (0.82–2.05)
High (>1500 KWD)	0.85 (0.58–1.24)	0.88 (0.60–1.29)
In direct contact with patients and provided medical care during COVID-19 pandemic		
Yes (Reference)	1.00	1.00
No	0.56 (0.33–0.96) (*p* = 0.033)	0.95 (0.55–1.63)
Specialty		
Physicians (Reference)	1.00	1.00
Pharmacists	1.11 (0.75–1.66)	1.03 (0.69–1.54)
Nurses	1.09 (0.47–2.50)	0.98 (0.44–2.20)
Allied health professionals	1.34 (0.83–2.16)	1.26 (0.80–1.97)

**Table 5:** Logistic regression analysis for undergraduate students in the health sciences center (HSCUs).

Variable	OR (95%CI) for Depression	OR (95%CI) for Anxiety
Gender		
Males (Reference)	1.00	1.00
Females	1.80 (0.85–3.83) (*p* < 0.01)	2.23 (0.83–6.00)
Age (Years)		
18–29	1.00	1.00
>29	0.26 (0.15–0.44) (*p* = 0.001)	0.43 (0.23–0.70)
Marital status		
Single (Reference)	1.00	1.00
Married	0.92 (0.44–1.91)	0.98 (0.43–2.21)
Divorced/Widowed	0.82 (0.05–13.17)	-
Stage of study		
Early years (Reference) *	1.00	1.00
Middle years *	1.09 (0.96–1.73)	1.18 (0.71–1.96)
Final years *	0.88 (0.55–1.43)	1.04 (0.61–1.76)
Chronic disease history		
Yes (Reference)	1.00	1.00
No	0.55 (1.03–1.94) (*p* = 0.018)	0.71 (0.36–1.38)

* Early years: students in 1st and 2nd years; Middle years: students in 3rd and 4th years; Final years: students in 5th, 6th, and 7th years.

## Data Availability

Not applicable.
